# ﻿A new species of *Habrophorula* from Vietnam and an updated key to species of the genus (Hymenoptera, Apidae)

**DOI:** 10.3897/zookeys.1197.118126

**Published:** 2024-04-18

**Authors:** Ngat Thi Tran, Michael S. Engel, Lien Thi Phuong Nguyen

**Affiliations:** 1 Institute of Ecology and Biological Resources, Vietnam Academy of Science and Technology, 18 Hoang Quoc Viet Road, Nghia Do, Cau Giay, Hanoi, Vietnam; 2 Division of Invertebrate Zoology, American Museum of Natural History, Central Park West at 79th Street, New York, New York 10024, USA; 3 Facultad de Ciencias Biológicas, Universidad Nacional Mayor de San Marcos, Lima, Peru; 4 Departamento de Entomología, Museo de Historia Natural, Universidad Nacional Mayor de San Marcos, Avenida Antonio Álvarez de Arenales 1256 Jesús María, Lima 14, Peru

**Keywords:** Anthophorini, Apoidea, morphology, new record, systematics, taxonomy, Vietnam

## Abstract

The rare bee genus *Habrophorula* Lieftinck, 1974 is recorded for the first time from Vietnam. The genus is represented by a new species, *Habrophorulabelladeceptrix* Tran, Engel & Nguyen, **sp. nov.**, from Cao Bang Province and can be most easily confused with *H.nigripes* Wu from China. The species is most easily differentiated by the unique form of the male terminalia but can also be distinguished by differences in integumental and setal coloration. A revised key is provided to the species of the genus. Females of the new species were collected at flowers of *Saurauiaroxburghii* Wall. and *Saurauianapaulensis* DC. (Actinidiaceae); males were collected at flowers of *Lantanacamara* L. (Verbenaceae).

## Abstract (vi)

Một giống ong hiếm có tên *Habrophorula* Lieftinck lần đầu tiên được ghi nhận ở Việt Nam. Trong giống này, *Habrophorulabelladeceptrix* Tran, Engel, & Nguyen, **sp. nov.** được phát hiện ở tỉnh Cao Bằng và loài này rất dễ nhầm lẫn với loài *H.nigripes* Wu từng được ghi nhận tại Trung Quốc. Chúng khác với các loài *Habrophorula* còn lại ở hình dạng độc đáo của bộ phận sinh dục đực, màu sắc của các đốt bụng và lông. Khoá định loại đến loài của giống này được cập nhật. Cá thể cái của loài ong này được thu thập trên hoa của loài Nóng roxburgh *Saurauiaroxburghii* Wall. và Nóng hoa nhọn *Saurauianapaulensis* DC. thuộc họ Dương đào (Actinidiaceae), cá thể đực được ghi nhận trên hoa của loài Bông ổi *Lantanacamara* L. thuộc họ Cỏ roi ngựa (Verbenaceae).

## ﻿Introduction

The uncommon southern Asian bee genus *Habrophorula* Lieftinck, 1974 belongs to the small *Elaphropoda*-group of the subfamily Anthophorinae (Michener 2007; Engel 2018; Orr et al. 2022). The genus is distinguished from others in this group by the following features: clypeus not greatly protuberant, extending anteriorly by about one-half compound eye width or less in profile; mandible tridentate; third submarginal cell about as wide on anterior margin as on posterior margin; hind leg of male not enlarged, metatrochanter lacking rounded projection; metasomal tergum VII and sternum VI of male not attenuate, apex of tergum VI nearly always bidentate or with emarginate apical truncation; sternum VII of male transverse, disc much broader than long, without apical process (Lieftinck 1974; Michener 2007). Individuals of *Habrophorula* are collected infrequently, and hitherto there have been only four species known, all from southern China (Wu 1991, 2000).

Here, *Habrophorula* is newly recorded for the first time from Vietnam, based on a series of females and males of a new species. We provided a description and figures for the new species along with a revised key for the identification of taxa in the genus.

## ﻿Materials and methods

Specimens examined in this study are deposited in the collection of Hymenoptera of the Institute of Ecology and Biological Resources (IEBR), Hanoi, Vietnam, and in the Division of Invertebrate Zoology, American Museum of Natural History, New York, New York (AMNH). Adult morphological and color characters were examined with a Nikon SMZ745 stereomicroscope, while images were photographed with a Nikon SMZ800N digital stereomicroscope, and with an attached ILCE-5000L/WAP2 digital camera. Stacked focus images were prepared using Helicon Focus 7. Lastly, all files were processed with Adobe Photoshop CS6. Male terminalia were dissected from relaxed specimens and then treated with Proteinase K so as to remove tissue and partially clear the integument. The morphological terminology used in the descriptions follows Engel (2001) and Michener (2007), with the following body metrics in mm (as used in Tran et al. 2022, 2023): **body length**: measured from the base of the antennal torulus to the metasomal apex (in dorsal view), **head length**: measured from the medioapical margin of the clypeus to the upper margin of the vertex (in facial view), **head width**: measured at the widest point of the head across the compound eyes (in facial view), **eye width**: the greatest width of the compound eye (in profile), **genal width**: the greatest width of the gena (in profile), **intertegular distance**: measured between the inner rims of the tegulae (in dorsal view). The abbreviations F, S, and T (followed by Arabic numerals) refer to numbered flagellomeres, metasomal sterna, and metasomal terga, respectively.

## ﻿Systematics

### ﻿Genus Habrophorula

Taxon classificationAnimaliaHymenopteraApidae

Lieftinck, 1974

3307E166-1534-582B-9D09-BABBA7F1C15A

Habrophorula Lieftinck, 1974: 217. Type species: Habropodanubilipennis Cockerell, 1930, original designation.

#### Note.

This is an uncommon genus that superficially resembles the more widely distributed *Elaphropoda* Lieftinck, species which are often more reddish in color. Unlike *Elaphropoda* the third submarginal cell is about as wide anteriorly as it is posteriorly (the cell is wider posteriorly in *Elaphropoda*); the clypeus is only moderately convex and not greatly protuberant, extending only about one-half the compound eye width, or less, in front of the compound eye when viewed in profile (except in the new species); male hind leg unmodified, metatrochanter lacking a rounded projection (hind leg of male enlarged, metatrochanter with broadly rounded projection in *Elaphropoda*); and tergum VII and sternum VI of the male not attenuate, apex of tergum VI of male with apical truncation weakly emarginate medially, and sternum VII of male with disc broader than long and lacking an apical process (tergum VII and sternum VI somewhat attenuate, tergum VI not emarginate, and sternum VII slightly broader than long and with apical process present in *Elaphropoda*) (Michener 2007; Engel 2018). Table 1 gives a summary of species currently included in *Habrophorula*, including species treated in this study, and with information on the known sexes and distribution.

**Table 1.** Summary of species currently in the genus *Habrophorula* Lieftinck (Anthophorini).

Species	Sexes known	Distribution
*Habrophorulabelladeceptrix* sp. nov.	♀♂	Vietnam (Cao Bang)
*Habrophorulaferruginipes* Wu, 1991	♂	China (Guangxi)
*Habrophorulanigripes* Wu, 1991	♀♂	China (Guizhou)
*Habrophorulanubilipennis* (Cockerell, 1930)	♀♂	China (Fujian, Hunan)
*Habrophorularubigolabralis* Wu, 2000	♀	China (Jiangxi)

Recently, Orr et al. (2022) treated the monotypic *Varthemapistra* Engel, 2008 as a synonym of *Habrophorula*. However, such a decision seems ill-advised at present, especially given that representatives of all anthophorine genera were included in the analysis with the exception of *Varthemapistra*, the sole specimen of which was also never examined by the authors. Engel (2018) did note that Varthemapistra might eventually be considered a subgenus of Habrophorula but in the absence of cladistic evidence for its placement as within or sister to *Habrophorula* or even a more inclusive clade of *Habrophorula* and other genera, any decision regarding its ultimate classification should await the discovery of additional specimens, particularly the currently unknown male. The simple female mandible of *Varthemapistraedentata* Engel, 2018 is not the result of wear as is easily evident from the holotype (which also does not show other signs of usual wear) and that both mandibles are identical (refer to Engel 2018: fig. 4 and discussion therein). The simple mandible is unique among anthophorines and, as such, while distinctive, is an autapomorphy. *Varthemapistra* was distinguished from *Habrophorula* and other anthophorines not only on the autapomorphic absence of mandibular teeth but also characters of the clypeus, metatibia, and especially forewing. It therefore seems groundless and unwarranted to place *V.edentata* in *Habrophorula* without cladistic evidence indicating the former renders the latter paraphyletic. Thus, given its unique suite of characters, its unique biogeographic occurrence relative to species of *Habrophorula*, and the possibility that it may be sister to *Habrophorula* (in which case its recognition as a genus or a distinctive subgenus serves to emphasize its unique characters and distribution), we retain the genus as distinct from *Habrophorula*.

### ﻿ Habrophorula belladeceptrix

Taxon classificationAnimaliaHymenopteraApidae

Tran, Engel & Nguyen sp. nov.

7FEA493F-DD76-5E9C-929F-2E1890E2C85D

https://zoobank.org/70DFD59A-1C27-4BF9-9097-30988B462051

Figs 1–2 , 3–6 , 7–10 , 11, 12 , 13–17 , 18–21 , 22–26

#### Diagnosis.

This species can be distinguished from among its congeners by the clypeus and supraclypeal area rather convex, extending in front of the compound eye almost as much as the compound eye width in profile, in this respect resembling the genus *Elaphropoda* (but can be distinguished from this genus by all of the other aforementioned characters). It could be easily confused with other species of *Habrophorula*, if ignoring the more protuberant clypeus, which have black legs and black apical margins of the metasomal terga, but differs in the fine clypeal markings of the female, the setal coloration of the male, and the male terminalia (Figs 18–21). Males could be confused with *H.nigripes* Wu except for the clypeal markings and setal coloration (*vide* key, *infra*) and, most notably, in the differences of the terminalia (*cf.*Wu 2000: fig. 186), particularly the forms of the hidden sterna. The terminalia somewhat resemble those of *H.nubilipennis* except in the new species sternum VII is narrower, sternum VIII is deeply concave medioapically, and the gonostylar setae are denser and more elongate.

#### Type material.

***Holotype*.** Vietnam: ♀, Cao Bang, Nguyen Binh, Phan Thanh, Salmon Station 2, Phia Oac−Phia Den National Park, 22°35′28′′N, 105°51′20′′E, alt. 1046 m, 2.vi.2023 [2 June 2023], NT Tran leg. (IEBR). ***Paratypes*.** Vietnam: 23♀♀, same data as holotype (IEBR, 2♀♀ AMNH); 2.vii.2022 [2 July 2022]; 1♀, Cao Bang, Nguyen Binh, Phan Thanh, Phia Oac-Phia Den NP, 22°35′03′′N, 105°51′40′′E, alt. 944 m, 9.vi.2020 [9 June 2020], LX Truong, LTP Nguyen, CQ Nguyen, HD Nguyen, NT Tran, TV Mai, UTP Tran leg.; 8♂♂, Nguyen Binh, Phan Thanh, Ca My Station of Resources Protection, Phia Oac−Phia Den National Park, 22°38′30′′N, 105°50′59′′E, alt. 1009 m, 7.vi.2020 [7 June 2020], LX Truong, LTP Nguyen, CQ Nguyen, HD Nguyen, NT Tran, TV Mai, UTP Tran leg. (IEBR, 2♂♂ AMNH); 18♂♂, alt. 1009 m, 3.vi.2023 [3 June 2023], NT Tran leg.

#### Description.

♀: Total body length 12 mm, forewing length 9 mm.

**Figures 1, 2.**
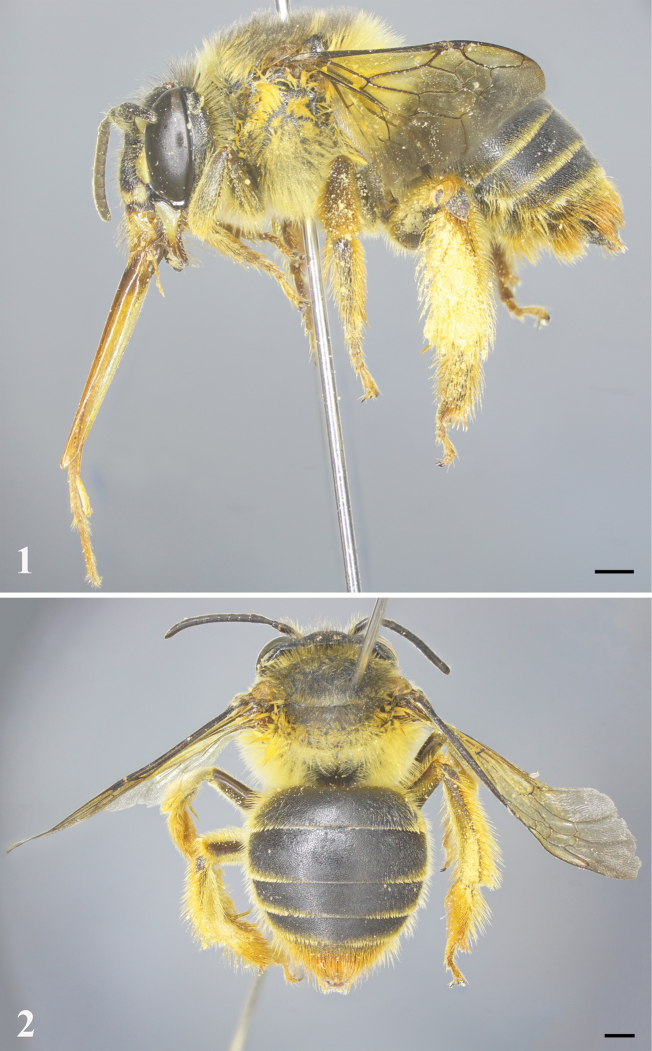
*Habrophorulabelladeceptrix* sp. nov., female **1** habitus, lateral view **2** habitus, dorsal view. Scale bars: 1 mm.

**Figures 3–6.**
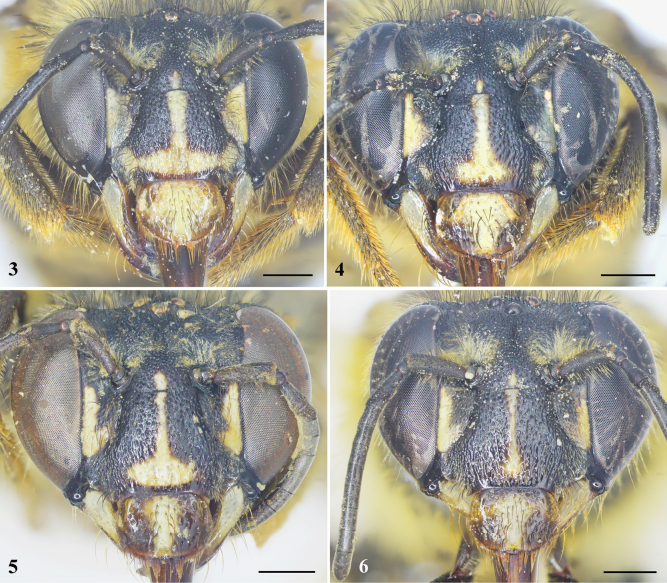
Facial marks variation of *Habrophorulabelladeceptrix* sp. nov., female **3** holotype **4–6** paratypes. Scale bars: 1 mm.

**Figures 7–10.**
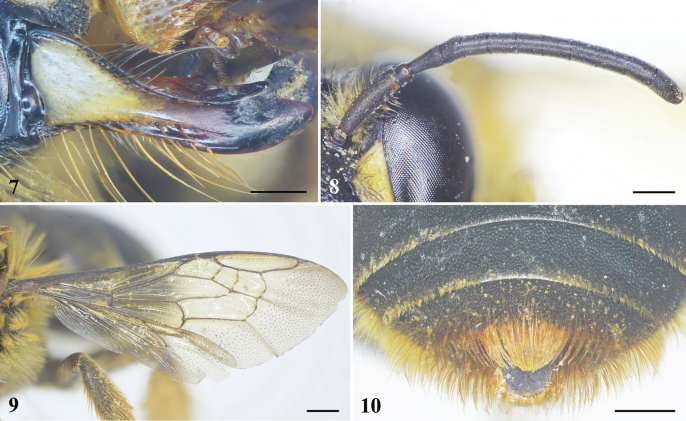
*Habrophorulabelladeceptrix* sp. nov., female **7** mandible, latero-dorsal view **8** left antenna **9** left forewing **10** pygidial plate, dorsal view. Scale bars: 0.5 mm (**7, 8**); 1 mm (**9, 10**).

***Structure*.** Head broader than long, about 1.4× as broad as long, head length 3.1 mm, width 4.4 mm (Fig. 3); compound eyes about 2× genal width; mandible with three teeth (as preserved, teeth relatively worn apically, and preapical tooth nearly completely worn) (Fig. 7); clypeus broader than long, about 1.5× as broad as long; clypeus and supraclypeal area rather convex, extending in front of compound eye almost as much as compound eye width in profile (unique to genus); labrum apically with small median emargination; scape slender, about 3.5× as long as broad, pedicel approximately 1.1× as broad as long and about 0.4× length of F1, F1 longer than broad and approximately 2× length of F2, F3–9 ascending in length, F3–5, F6–7, and F8–9 subequal in length, F10 longest flagellomere, about 1.4× as long as broad (Fig. 8). Mesosoma approximately as broad as long; mesoscutellum short and with apical margin rounded, not overhanging metanotum (Fig. 2). Forewing with three submarginal cells, first and third submarginal cells broader than second submarginal cell, 1m-cu entering near apex of second marginal cell (Fig. 9). Metabasitibial plate large (Fig. 1). Metasoma rather heart-shaped (Fig. 2); pygidial plate large, rounded apically (Fig. 10).

***Sculpturing and texture*.** Clypeus and supraclypeal area with dense, coarse punctures, such punctures becoming elongate apically to give surface a wrinkled appearance (Fig. 3); paraocular area below antennal torulus with punctures similar to elongate punctures of clypeus except shallow and more spaced; frons and vertex with small, round, dense punctures separated by less than a puncture width, such punctures in ocellocular area becoming sparse, integument between punctures smooth; gena with dense, elongate punctures. Mesoscutum and mesoscutellum with large, round, dense punctures separated by much less than a puncture width; metanotum with punctures similar to mesoscutellum except smaller, such punctures becoming fainter mesally and integument more imbricate; pleura with punctures similar to mesoscutum except contiguous; basal area of propodeum wholly vertical, scarcely differentiated from posterior surface, with punctures similar to mesoscutellum on basal area and lateral and posterior surfaces. Metasomal terga with small, round, punctures separated by a puncture width, integument between smooth and matte, such punctures denser in apical marginal zones and progressively so laterally on each tergum and on more apical terga; sterna with coarser punctures than those of terga, separated by less than a puncture width, smaller and denser toward apical marginal zones, narrow apical margins impunctate, pregradular surfaces impunctate and imbricate.

***Color*.** Labrum brown, except yellowish mark medially (Fig. 3); mandible with yellow mark basally, then brown to black on remainder (Fig. 7); paraocular area with yellowish marking extending along inner margin to level of antennal toruli, except black spot on upper side of clypeus; clypeus black, except inverted yellowish T-shaped mark medially and apically, and brown mark apically; supraclypeal area with small yellowish mark medially (Fig. 3). Yellowish marks of labrum, clypeus and paraocular area variety in paratypes (Figs 4–6). Remainder of integument black.

***Pubescence.*** Clypeus with some short, yellowish setae intermixed with black setae latero-apically; paraocular area with short, yellowish setae intermixed with sparse black setae; scape with long, brownish black setae; face above antennal torulus with yellowish tuft of setae (Fig. 3); vertex with long, blackish setae; occiput with long, yellowish, dense setae. Mesosoma with long, dense, yellowish setae intermixed with blackish setae anteriorly and yellowish setae laterally (Figs 1, 2), such setae sparse to absent on disc. Coxae and trochanters with dense, yellowish setal tufts ventrally; outer surface of mesotibia and mesobasitarsus with long, yellowish-orange setae, inner surface of mesobasitarsus with short, dense, orange setae; outer surface of metatibia and metabasitarsus with yellowish-orange scopal setae. Apical margins of metasomal T1–3 with short, yellowish setal bands, interrupted medially; T4 with short setal band apically; T5 with long, dense, orange setae (Fig. 2); T6 covered with orange setae lateral to pygidial plate (Fig. 10); S2–4 apical margins with long, sparse, yellowish-orange setae; S5 apical margin with long, dense, orange setae; S6 apically with orange setal tufts.

♂: Total body length about 10 mm, forewing length 9 mm. Head in facial view with yellowish marks as in Figs 13–15; mandible with three teeth, prominent preapical tooth and two long apical teeth (Fig. 14); antenna with scape about 2.5× as long as broad, F1 approximately 0.8× length of F2, F3–10 subequal in length, F11 longest flagellomere (Fig. 15); forewing as in Fig. 16; T7 with apical margin concave medially to form short, broad, paramedial lobes (Fig. 17). Male terminalia as in Figs 18–21.

Sculpturing as described for female (*vide supra*) except coarse punctures of sterna sparser.

Integument black except mandible largely yellow with black apex, labrum yellow with basolateral ovals of semitranslucent brown; clypeus with large, inverted-T-shaped yellow marking; paraocular area below antennal torulus pale yellow to off-white, somewhat diaphanous; venter of scape with yellow longitudinal stripe.

**Figures 11, 12.**
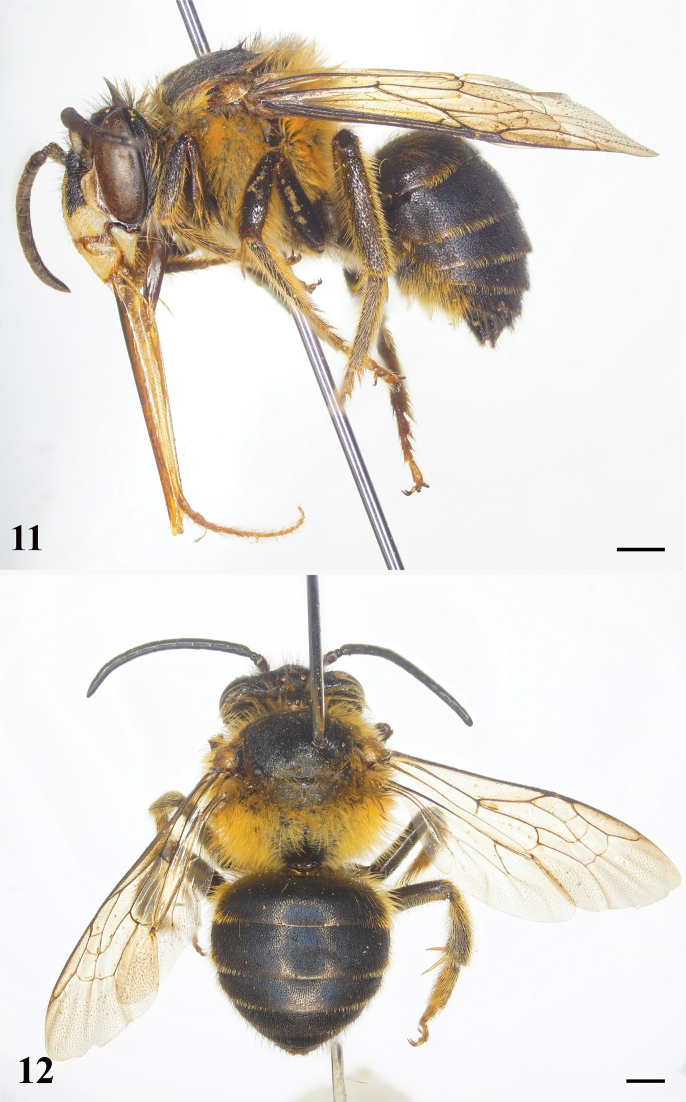
*Habrophorulabelladeceptrix* sp. nov., male **11** habitus, lateral view **12** habitus, dorsal view. Scale bars: 1 mm.

Metasomal T1 basally and laterally with relatively long, yellowish-orange setae; T1–T5 apically with short, yellowish-orange to yellowish setal bands, broadly interrupted medially (Figs 11, 12).

**Figures 13–17.**
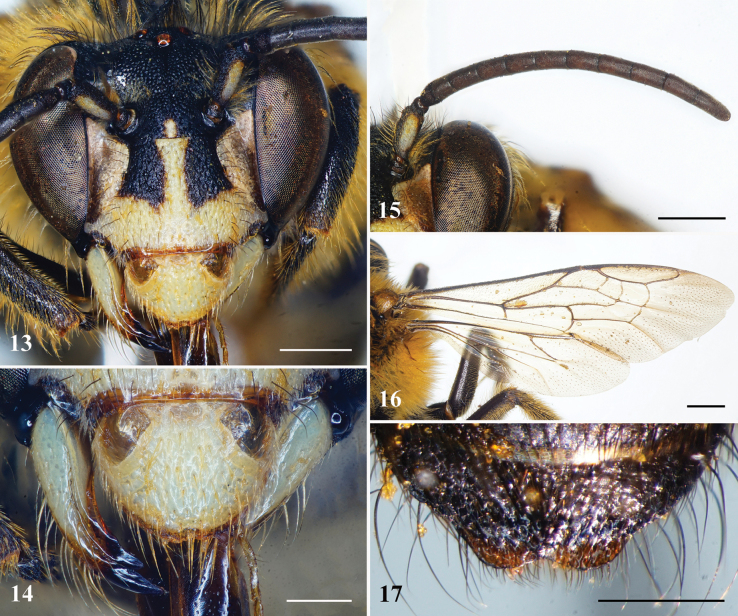
*Habrophorulabelladeceptrix* sp. nov., male **13** head, facial view **14** labrum and mandible, dorsal view **15** left antenna. **16** right forewing **17** metasomal tergum VII, dorsal view. Scale bars: 0.5 mm (**14, 17**); 1 mm (**13, 15, 16**).

**Figures 18–21.**
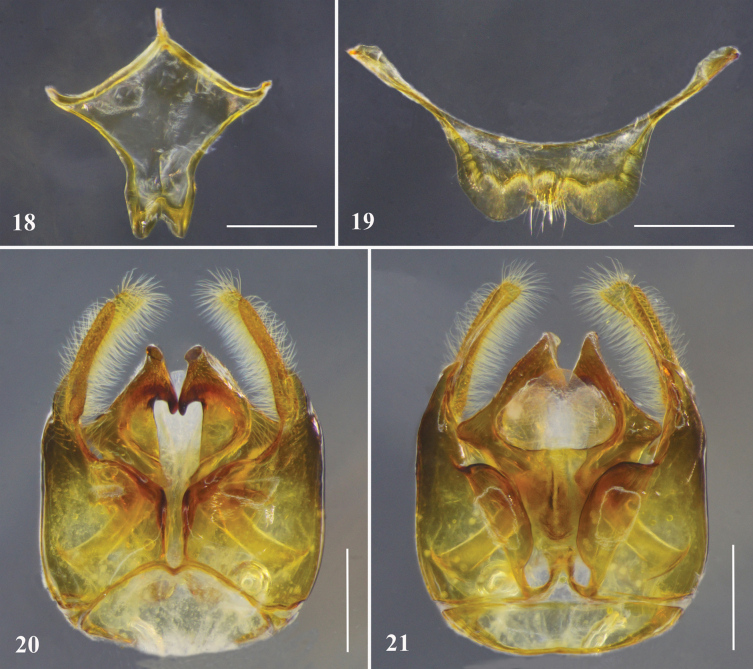
*Habrophorulabelladeceptrix* sp. nov., male terminalia **18** metasomal sternum VII **19** metasomal sternum VIII **20** genitalia, dorsal view **21** genitalia, ventral view. Scale bars: 0.5 mm.

#### Etymology.

The specific epithet is a combination of the Latin adjectives *bellā*, meaning, “beautiful”, and *dēceptrix*, meaning, “she who deceives”.

#### Remarks.

**Figures 22–26.**
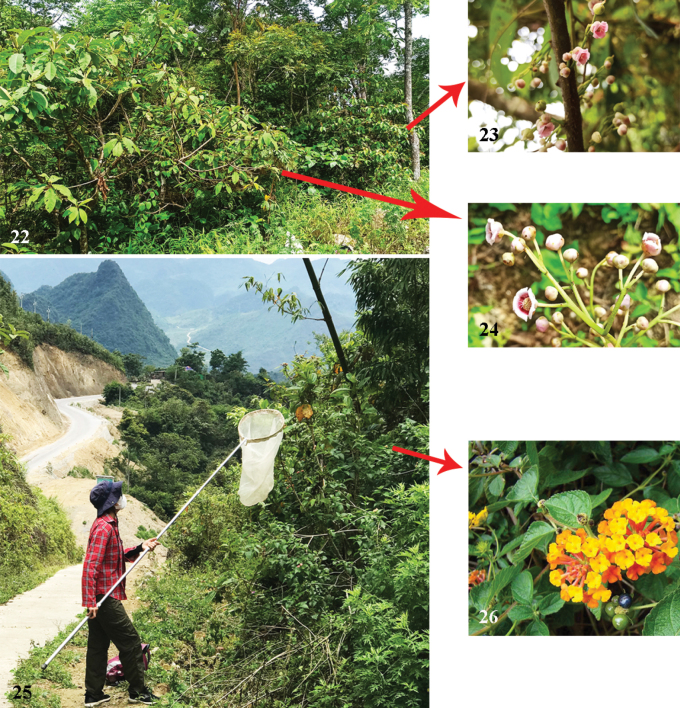
Habitat and floral associations of *Habrophorulabelladeceptrix* sp. nov. in Vietnam **22** habitat where females were found **23** flowers of *Saurauiaroxburghii* Wall. (Actinidiaceae) at which females were collecting **24** flowers of *Saurauianapaulensis* DC. (Actinidiaceae) at which females were collected **25** habitat where males were found **26** flowers of *Lantanacamara* L. (Verbenaceae).

This species was collected exclusively from Phia Oac-Phia Den National Park, Cao Bang Province (Figs 22, 25). Females were collected from flowers of *Saurauiaroxburghii* Wall. (Fig. 23) and *Saurauianapaulensis* DC. (Actinidiaceae) (Fig. 24), which are relatively common on the sides of roads. Its associated sex was recorded from flowers of *Lantanacamara* L. (Verbenaceae) (Fig. 26).

### ﻿Revised key to species of *Habrophorula*

The following key is expanded and revised from that presented by Wu (2000). Characteristics for the key were extracted from the original descriptions of the species (Lieftinck 1974; Wu 1991, 2000), and the examination of identified species in the Division of Entomology, University of Kansas Natural History Museum. The male of *H.rubigolabralis* and the female of *H.ferruginipes* remain unknown.

**Table:** 

1	Females	**2**
–	Males	**5**
2(1)	Metasomal T1–4 with apical margins black; body lengths 10.5–13 mm	**3**
–	Metasomal T1–4 with apical margins broadly reddish brown; body lengths 13–14 mm	***H.nubilipennis* (Cockerell)**
3(2)	Legs black; clypeus variable, but markings never reddish brown, instead yellow	**4**
–	Legs brown; clypeus black with reddish brown inverted T-shaped mark medially and apically, apical portion long and with mountain-peak-shaped extensions on either side of middle stripe (see Wu 2000: fig. 187); body length 10.5 mm	***H.rubigolabralis* Wu**
4(3)	Clypeus black with thin, yellow inverted T-shaped mark medially and apically; body length 12 mm	***H.belladeceptrix* sp. nov.**
–	Clypeus largely yellow, with large extensions of black basolaterally and extending to about two-thirds length (see Wu 2000: fig. 186g); body lengths 12–13 mm	***H.nigripes* Wu**
5(1)	Legs yellow to testaceous or reddish brown; clypeus with yellow markings; mesosoma with yellow or griseous setae	**6**
–	Legs black; clypeus with yellow or cream white markings; mesosoma covered with yellow or tawny setae; body length 10 mm	**7**
6(5)	Legs and tegula reddish brown, antenna reddish brown; mesosoma covered with griseous setae; body length 12 mm	***H.nubilipennis* (Cockerell)**
–	Legs and tegula yellow, antenna dark brown; mesosoma covered with yellow setae; body length 10 mm	***H.ferruginipes* Wu**
7(5)	Clypeus with yellow markings; mesosoma with yellow setae except more tawny on pleura and propodeum	***H.belladeceptrix* sp. nov.**
–	Clypeus with cream white markings; mesosoma with yellow setae	***H.nigripes* Wu**

## Supplementary Material

XML Treatment for Habrophorula

XML Treatment for Habrophorula belladeceptrix

## ﻿Additional information

### Author ORCIDs

Ngat Thi Tran https://orcid.org/0000-0002-7057-4638

Michael S. Engel https://orcid.org/0000-0003-3067-077X

Lien Thi Phuong Nguyen https://orcid.org/0000-0003-3527-9577

### Data availability

All of the data that support the findings of this study are available in the main text.
